# Diverse captive non-human primates with phytanic acid-deficient diets rich in plant products have substantial phytanic acid levels in their red blood cells

**DOI:** 10.1186/1476-511X-12-10

**Published:** 2013-02-04

**Authors:** Ann B Moser, Jody Hey, Patricia K Dranchak, Mazen W Karaman, Junsong Zhao, Laura A Cox, Oliver A Ryder, Joseph G Hacia

**Affiliations:** 1 Department of Neurogenetics, Hugo W. Moser Research Institute at Kennedy Krieger, Baltimore, MD, 21205, USA; 2 Department of Genetics, Rutgers University, Piscataway, NJ, 08854, USA; 3 Department of Biochemistry and Molecular Biology, Broad Center for Regenerative Medicine and Stem Cell Research, University of Southern California, Los Angeles, CA 90089, USA; 4 Department of Genetics, Southwest National Primate Research Center, Texas Biomedical Research Institute, San Antonio, TX, 78227, USA; 5 Institute for Conservation and Research, Zoological Society of San Diego, Escondido, CA, 92027, USA

**Keywords:** Phytanic acid, Chlorophyll, Old world monkeys, New world monkeys, Peroxisome

## Abstract

**Background:**

Humans and rodents with impaired phytanic acid (PA) metabolism can accumulate toxic stores of PA that have deleterious effects on multiple organ systems. Ruminants and certain fish obtain PA from the microbial degradation of dietary chlorophyll and/or through chlorophyll-derived precursors. In contrast, humans cannot derive PA from chlorophyll and instead normally obtain it only from meat, dairy, and fish products.

**Results:**

Captive apes and Old world monkeys had significantly higher red blood cell (RBC) PA levels relative to humans when all subjects were fed PA-deficient diets. Given the adverse health effects resulting from PA over accumulation, we investigated the molecular evolution of thirteen PA metabolism genes in apes, Old world monkeys, and New world monkeys. All non-human primate (NHP) orthologs are predicted to encode full-length proteins with the marmoset *Phyh* gene containing a rare, but functional, GA splice donor dinucleotide. *Acox2*, *Scp2*, and *Pecr* sequences had amino acid positions with accelerated substitution rates while *Amacr* had significant variation in evolutionary rates in apes relative to other primates.

**Conclusions:**

Unlike humans, diverse captive NHPs with PA-deficient diets rich in plant products have substantial RBC PA levels. The favored hypothesis is that NHPs can derive significant amounts of PA from the degradation of ingested chlorophyll through gut fermentation. If correct, this raises the possibility that RBC PA levels could serve as a biomarker for evaluating the digestive health of captive NHPs. Furthermore, the evolutionary rates of the several genes relevant to PA metabolism provide candidate genetic adaptations to NHP diets.

## Background

**Figure 1:**
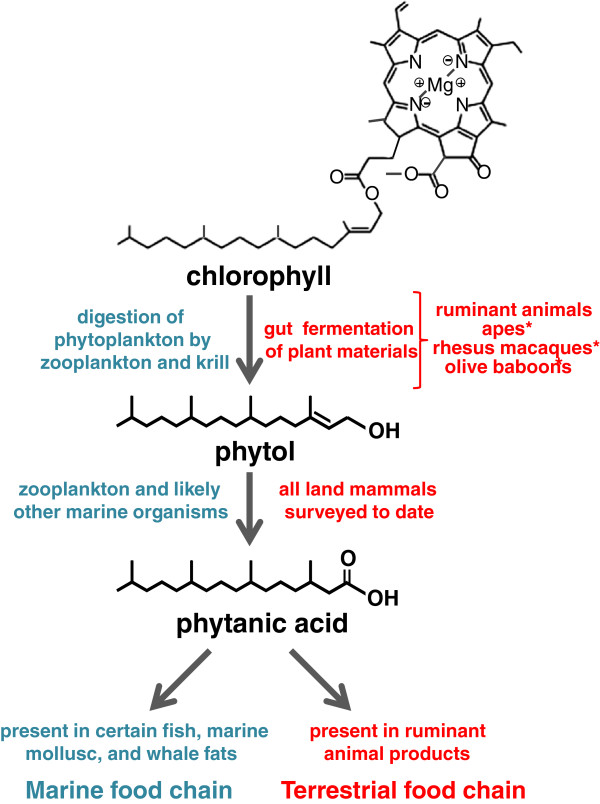
**Phytanic acid in the food chain. **Simplified schematic representation of the mechanism by which phytanic acid is introduced into marine and terrestrial food chains. Asterisks next to the apes (including bonobos, chimpanzees, gorillas, orangutans, and siamangs), rhesus macaques, and olive baboons indicate they are proposed to degrade chlorophyll into phytol based on data from previous studies [3] as well as this study. Interactions from the marine food chain are based, in part, on reference [2]. Although chlorophyll A is depicted, the same pathway also pertains to chlorophyll B.

Phytanic acid (PA) is a branched chain fatty acid that can be acquired in some species by ingesting plant and/or animal products [1,2] (Figure 1). In ruminants, the fermentation of ingested plant materials by gut microbes can liberate phytol, a constituent of chlorophyll, which can be rapidly metabolized to PA and stored in fats [1,2]. Members of the marine food chain can accumulate PA by ingesting zooplankton and/or krill, sources of phytol and chlorophyll-related precursors [2]. Although humans can convert free phytol into PA, they do not derive appreciable amounts of PA from chlorophyll in plant materials, but can obtain PA from the ingestion of ruminant fats, fish, and dairy products [3]. In contrast, captive great apes (chimpanzees, bonobos, gorillas, and orangutans) maintained on PA-deficient diets have substantial PA levels in their red blood cells (RBCs) [3]. It was proposed that these animals derived PA from microbial degradation of ingested chlorophyll during hindgut fermentation [3].

Although, there is no evidence that the PA or phytol content of normal diets effects human health, humans [2,4] and rodents [5] with severely impaired PA metabolism have increased circulating PA levels and accumulate PA stores. In humans, this results in Refsum disease, which typically manifests as a peripheral polyneuropathy with cerebellar ataxia and loss of vision, hearing and olfaction [2,4]. Thus, strong selective pressure could exist to maintain efficient PA metabolism in organisms that can obtain it from dietary sources [3,6].

Here, we explored the hypothesis that diverse non-human primates (NHPs) can obtain PA from plant materials and accumulate it in their cells. In parallel, we investigated the molecular evolution of PA metabolism genes in NHPs. These biochemical and genetic data provide evidence there is selective pressure to retain PA metabolism in diverse NHPs as a result of their ability to obtain this potentially toxic branched chain fatty acid from plant materials.

## Materials and methods

We obtained University of Southern California Institutional Review Board approval for human subjects research. Whole blood samples from fasting donors were stored and analyzed by capillary GC–electron-capture negative-ion mass spectrometry [3]. All NHPs, except common marmosets, should have <1 milligram PA intake daily [7]. Since the marmoset diet includes low-fat yogurt as a supplemental enrichment item and, more rarely, low-fat cottage cheese, it is not classified as being PA-deficient. Fresh vegetables and fruits typically available in Western grocery stores have minimal phytol levels [7]. Additional file 1 and Additional file 2 provide donor information and PA measurements, respectively.

Additional file 3 and Additional file 4 respectively provide DNA sequences and aligned protein sequences and the data mining and sequencing methods used to acquire them [8-10]. The methods and results for estimating gene phylogenies and testing for variation in the ratios of non-synonymous to synonymous (dN/dS) mutation rates are provided in Additional file 5.

## Results

**Figure 2:**
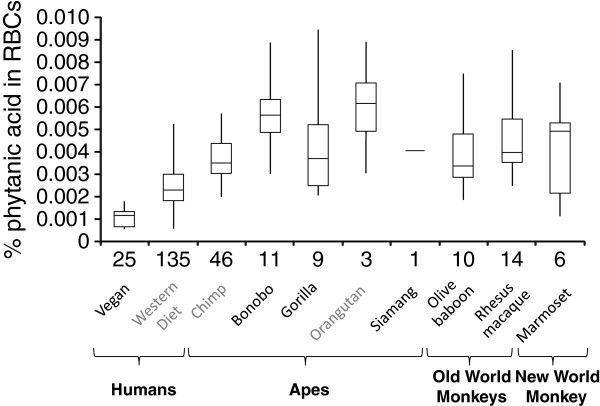
**Phytanic acid levels in non-human primate (NHP) red blood cells (RBCs). **Box plots representing the percentage of phytanic acid relative to total fatty acids from RBCs are provided. Median, quartile 1, quartile 3, minimum, and maximum values are provided. The number of individuals for each species is provided on the X-axes. These are annotated in black font if the information is partially (human vegan, bonobo, and gorilla) or completely (olive baboon, rhesus macaque, common marmoset, and siamang) newly generated for this manuscript (Additional file 2). The data annotated in gray font was previous reported by ourselves using the same methods [3]. Combined data from males and females are used in this analysis.

**Figure 3:**
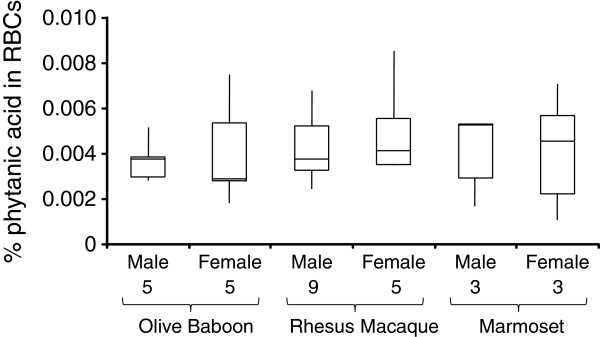
**Phytanic acid levels in NHP RBCs by sex. **Box plots representing the percentage of phytanic acid relative to total fatty acids from RBCs are provided from male and female olive baboons, rhesus macaques, and common marmosets. Median, quartile 1, quartile 3, minimum, and maximum values are provided. The gender, number of individuals successfully analyzed, and species are provided on the X-axis.

Human vegans had lower RBC PA levels than the NHP cohort (*P*<1×10^-10^). All NHP groups on PA-deficient diets had elevated RBC PA levels (*P*<1×10^-7^) relative to vegans (chimpanzees 3.5-fold; bonobo 5.6–fold; gorillas 3.9–fold; olive baboons 3.7-fold; rhesus macaques 4.3-fold) (Figure 2). The single siamang had 4.1–fold elevated RBC PA levels relative to vegans (Figure 2). Although marmosets had 1.5-fold elevated RBC PA levels relative to vegans (*P*<1×10^-5^), the levels in 2/6 marmosets were within the vegan range (Additional file 2). All NHP groups on PA-deficient diets had ≥1.5-fold increased PA levels (*P*<1×10^-3^) to humans on Western diets with 50–100 mg daily PA intake [4]. Consistent with prior reports in humans [3], significant sex-specific differences (*P*<0.05) were not found in rhesus macaques, olive baboons, and marmosets (Figure 3). This contrasts with sexual dimorphism observed in chimpanzee and mouse RBC PA levels [3]. A male and female marmoset had PA levels comparable to vegans while two male and two female marmosets had PA levels comparable to great apes.

We obtained complete orthologous exon sequences for 13 critical PA metabolism genes [3] in great apes as well as representative lesser apes, Old World and New World monkeys (Additional file 3). All 136 complete NHP protein sequences were predicted to be full length relative to human (Additional file 4). The PTS1 peroxisome targeting sequence motifs of the *Acox2*, *Acox3*, *Ehhadh*, *Scp2* , and *Pecr* genes were predicted to be functional in all NHPs using the PTS1 predictor tool [11] (Additional file 5). The *Amacr* PTS1 signal was predicted to be functional in all NHPs except marmoset, where it had questionable predicted activity (Additional file 5). The *Phyh* and *Acaa1* PTS2 peroxisome targeting sequence motifs matched the reported consensus sequence in all NHPs (Additional file 5) [12].

We encountered two instances of noncanonical splice donor/accepter dinucleotide sequences in at least one species. A noncanonical *Phyh* 5’-GA splice donor is present in marmosets and Bolivian squirrel monkeys (BSM). Based on a newly obtained marmoset *Phyh* liver cDNA sequence, we found this 5’-GA splice donor [13] is functional. The BSM *Scp2* intron 13 sequence had a noncanonical 3’-AC splice acceptor sequence that, in principle, could be functional.

*Acox2* (log-likelihood ratio statistic (LLR)=9.638, *P*=0.0081, 2 degrees of freedom (df)), *Scp2* (LLR=6.049, *P*=0.0485, 2df), and *Pecr* (LLR=16.111, *P*=0.00032, 2df) showed clear evidence of having amino acid positions with dN/dS values >1 in primates (Additional file 6). A Bayes-Empirical-Bayes (BEB) analysis [14] identified one position (human residue arginine614) in Acox2, two positions (human residues proline5 and serine125) in Scp2, and 3 positions in Pecr (human residues proline160, tryptophan216, and glycine223) with a >95% chance of having a dN/dS value >1. We asked whether dN/dS values varied significantly over the phylogeny, by allowing for separate dN/dS rates on the clade of ape sequences compared to the rest of the tree. *Amacr* showed statistically significant variation in dN/dS values in this analysis (LLR=40.349, *P*=0.00984, 22df). None of the branch-site tests, in which the branch leading to the apes was contrasted with the remainder of the phylogeny, showed significant variation in dN/dS values. Similarly for the branch-site analyses in which the human branch was allowed to vary with respect to the rest of the tree in dN/dS value, no loci showed a significant departure from the null model.

We excluded the gorilla *Amacr* gene for our analyses since it is partially duplicated in the gorilla genome [15]. Although the gorGor3.1/gorGor3 gorilla genome assembly predicts a premature UGA (stop) codon at position 208, we found evidence of a CGA (arginine) codon in sequence traces from ten different gorillas. Reanalysis of RNA-Seq analysis from gorilla tissues [9] confirmed the CGA codon, which suggests that the gorilla *Amacr* gene encodes a full length protein.

## Discussion

Relative to humans, diverse NHPs have higher RBC phytanic levels when fed plant-rich PA-deficient diets. Ruminants can acquire PA from the microbial degradation of chlorophyll through gut fermentation, which liberates phytol side chains that mammals can convert to PA [16]. Great apes and other primates rely more heavily upon gut fermentation to meet their metabolic energy needs than do humans [3,17]. As such, the favored hypothesis is that, unlike humans, diverse NHPs can obtain substantial amounts of PA through the microbial degradation of ingested chlorophyll during gut fermentation. If correct, RBC PA levels could provide a biomarker of gut fermentation activity useful for evaluating the digestive health of diverse NHPs.

Alternative hypotheses include the increased retention of PA in NHPs relative to humans [3]. Given the minimal dietary PA acid exposure in our cohort fed PA-deficient diets, even when taking body weights into consideration, this would require extreme differences in retention rates. Rigorous testing of these hypotheses would require measuring the metabolism of ingested radiolabeled chlorophyll, phytol, and/or phytanic acid [18]; however, ethical issues preclude these experiments from being performed. The fact that human and great ape cultured fibroblasts all have robust PA metabolic activity [3] suggests that the higher RBC PA levels in great apes not primarily due to cross-species differences in PA metabolic activity.

Our observations raised the possibility that the molecular evolution rates of NHP genes relevant to the pharmacokinetics of PA, chlorophyll, and chlorophyll metabolites could be influenced by selective pressure. Prior analysis using partial coding sequences suggested that a group of nine peroxisome genes evolved under positive selection in the ancestral primate lineage [19]. Five (*Pex7*, *Hacl1*, *Scp2*, *Acox3*, and *Phyh*) are directly relevant to PA metabolism. Here, we present evidence that *Amacr* evolutionary rates varied across primate lineages and that *Acox2*, *Scp2*, and *Pecr* have amino acid positions with accelerated substitution rates. The functional significance of these amino acid substitutions warrants more in-depth biochemical analysis. Although there was no clear evidence of compromised NHP PA metabolic activities, the intracellular localization of Amacr could differ in marmosets relative to other primates.

## Conclusions

Relative to humans, diverse NHPs on PA-deficient diets rich in plant materials have elevated RBC PA levels. Four genes relevant to PA metabolism showed varied evolutionary rates across primate lineages or amino acid positions with accelerated substitution rates. These genes, and likely others relevant to PA homeostasis [20], provide additional candidate adaptations to NHP diets [6,19,21-25]. Additional analyses of RBC and plasma lipids [25] could highlight additional biochemical pathways under selection in primate lineages.

## Competing interests

The authors declare that they have no competing interests.

## Authors’ contributions

ABM carried out all the biochemical analyses and was involved in the design and conception of the project. JH conducted all the statistical tests for selection and helped in the writing of the manuscript. OAR provided great ape blood samples and diet information and was involved in the design and conception of the project. PKD, MWK, and JZ helped acquire NHP gene sequence information. LAC provided baboon, marmoset and rhesus blood samples and diet information, baboon sequence data and edited the manuscript. JGH was involved in the overall design and conception of the project, statistical analysis of all data sets, and wrote the manuscript with the help of all authors. All authors read and approved the final manuscript.

## Supplementary Material

Additional file 1 **Composition of blood donor cohort. **A summary of the numbers, ages, and sex of blood donors is provided. Click here for file

Additional file 2 **Phytanic acid relative to total fatty acid levels for each red blood cell donor.** Relative levels of phytanic acid for all RBC donors are provided. (XLSX 16 kb) Click here for file

Additional file 3 **Orthologous gene sequences used in this study. **All orthologous gene sequences and the methods used to acquire them are provided. Click here for file

Additional file 4 **Aligned protein sequences used in this study.** Aligned protein sequences for all ten genes interrogated in this study are provided. Click here for file

Additional file 5 **Orthologous NHP peroxisome targeting sequences (PTS1 or PTS2) for peroxisome matrix proteins. **Orthologous NHP peroxisome targeting sequences (PTS1 or PTS2) for peroxisome matrix proteins are provided. Click here for file

Additional file 6 **Results of PAML likelihood ratio tests for selection. **Results of PAML likelihood ratio tests for selection are provided. Click here for file
