# Supplementary material for: Diverse captive non-human primates with phytanic acid-deficient diets rich in plant products have substantial phytanic acid levels in their red blood cells
Source: Lipids Health Dis. 2013 Feb 4;12:10. doi: 10.1186/1476-511X-12-10 (PMC3571895; doi:10.1186/1476-511X-12-10)
Supplement: Additional file 4 — Aligned protein sequences used in this study. Aligned protein sequences for all ten genes interrogated in this study are provided. [file 1476-511X-12-10-S4.pdf]

PHYH

```
                    10        20        30        40        50        60        70        80        90
                     |         |         |         |         |         |         |         |         |
Human       MEQLRAAARLQIVLGHLGRPSAGAVVAHPTSGTISSASFHPQQFQYTLDNNVLTLEQRKFYEENGFLVIKNLVPDADIQRFRNEFEKICR
Chimpanzee  ...P.........................I............................................................
Bonobo      ...P.........................I............................................................
Gorilla     ...P......................................................................................
Orangutan   ...P......................................................................................
Siamang     ...P......................................................................................
Gibbon      ...P...........................................................................C..........
IRMacaque   ...P..........................T...A.......................................................
CRMacaque   ...P..........................T...A.......................................................
CEMacaque   ...P..........................T...A.......................................................
LTMacaque   ...P..........................T...A.......................................................
OBaboon     ...P........I....R............T...........................N...............................
GBaboon     ...P..........................T.......I...................................................
DLangur     ...S......................V...T...........................................................
Marmoset    ...S...S..R.......S...LNI.G......V............S..................Y.......S................
BSqMonkey   ...S..................R.I.G...................S..................Y.......S................

                   100       110       120       130       140       150       160       170       180
                     |         |         |         |         |         |         |         |         |
Human       KEVKPLGLTVMRDVTISKSEYAPSEKMITKVQDFQEDKELFRYCTLPEILKYVECFTGPNIMAMHTMLINKPPDSGKKTSRHPLHQDLHY
Chimpanzee  ..........................................................................................
Bonobo      ..........................................................................................
Gorilla     .........I................................................................................
Orangutan   ..........................................................................................
Siamang     ..........................................................................................
Gibbon      .....................V....................................................................
IRMacaque   .....F..V............T.....T..............................................................
CRMacaque   .....F..V............T.....T..............................................................
CEMacaque   .....F..V............T.....T..............................................................
LTMacaque   .....F..V............T.....T..............................................................
OBaboon     .....F..V............T.....T..............................................................
GBaboon     .....F..V............T.....T..............................................................
DLangur     ........V..................T..............................................................
Marmoset    ..............A.L.....H...TV..............................................A...............
BSqMonkey   ..............A.L....PHG....A......D.Q....................................A...............

                   190       200       210       220       230       240       250       260       270
                     |         |         |         |         |         |         |         |         |
Human       FPFRPSDLIVCAWTAMEHISRNNGCLVVLPGTHKGSLKPHDYPKWEGGVNKMFHGIQDYEENKARVHLVMEKGDTVFFHPLLIHGSGQNK
Chimpanzee  ...................N......................................................................
Bonobo      ...................N......................................................................
Gorilla     ...................N......................................................................
Orangutan   ...................D........................................D.NT..........................
Siamang     ...................D......................................................................
Gibbon      ...................D....................N..................K..............................
IRMacaque   ......N............D..........................................N...........................
CRMacaque   ......N............D..........................................N...........................
CEMacaque   ......N............D..........................................N...........................
LTMacaque   ......N............D..........................................N...........................
OBaboon     ......N............D..........................................N...........................
GBaboon     ......N............D..........................................N...........................
DLangur     ......N............D..........................................N...........................
Marmoset    ......N..........P.D.............R............................T........................R..
BSqMonkey   ......N..........P.D.............R............................T........................R..

                   280       290       300       310       320       330
                     |         |         |         |         |         |
Human       TQGFRKAISCHFASADCHYIDVKGTSQENIEKEVVGIAHKFFGAENSVNLKDIWMFRARLVKGERTNL
Chimpanzee  ................................................D...................
Bonobo      ................................................D...................
Gorilla     ................................................D...................
Orangutan   .........................................Y......D...................
Siamang     ..................................L...N.LY......D...................
Gibbon      ......................................N.LY......D...................
IRMacaque   ......................................N..W.D...MD................I..
CRMacaque   ......................................N..W.D...MD................I..
CEMacaque   ......................................N..W.D...MD................I..
LTMacaque   ......................................N..W.D...MD................I..
OBaboon     ......................................N..W.D...M.................I..
GBaboon     ......................................N..W.D...MD................I..
DLangur     ......................................N..Y.....TD......Y............
Marmoset    ....................N.............A.L.N.LY.LAD..D.................S.
BSqMonkey   ....................N............IA.L.N.LY.LD.T.D...................
```

Green font highlighted in yellow: Human and Neandertal amino acid residue differ from chimpanzee.

HACL1

```
                     10        20        30        40        50        60        70        80        90
                      |         |         |         |         |         |         |         |         |
Human        MPDSNFAERSEEQVSGAKVIAQALKTQDVEYIFGIVGIPVTEIAIAAQQLGIKYIGMRNEQAACYAASAIGYLTSRPGVCLVVSGPGLIH
Chimpanzee   ..........................................................................................
Gorilla      ..........................................................................................
Orangutan    ..........................................................................G...............
Gibbon       ..........Q.P.............................................................G...............
IRMacaque    ...............................V..........................................G...............
CRMacaque    ...............................V..........................................G...............
CEMacaque    ...............................V..........................................G...............
OBaboon      ...............................V..........................................G...............
Marmoset     ..........................................................................G...............
BSqMonkey    .............M.................M..........................................G...............

                    100       110       120       130       140       150       160       170       180
                      |         |         |         |         |         |         |         |         |
Human        ALGGMANANMNCWPLLVIGGSSERNQETMGAFQEFPQVEACRLYTKFSARPSSIEAIPFVIEKAVRSSIYGRPGACYVDIPADFVNLQVN
Chimpanzee   ..........................................................................................
Gorilla      ..........................................................................................
Orangutan    ..........................................................................................
Gibbon       ..........................................................................................
IRMacaque    .........V...........P..................................V.................................
CRMacaque    .........V...........P.....M............................V.................................
CEMacaque    .........V...........P..................................V.................................
OBaboon      .........V...........P..................................V.................................
Marmoset     ................M.................................................T.......................
BSqMonkey    ..................................................................T.......................

                    190       200       210       220       230       240       250       260       270
                      |         |         |         |         |         |         |         |         |
Human        VNSIKYMERCMSPPISMAETSAVCTAASVIRNAKQPLLIIGKGAAYAHAEESIKKLVEQYKLPFLPTPMGKGVVPDNHPYCVGAARSRAL
Chimpanzee   ..........................................................................................
Gorilla      ........................M.................................................................
Orangutan    ...............................................................................C..........
Gibbon       ..............V............................................C..............................
IRMacaque    .S...HV.......................K......................Q....................................
CRMacaque    .S...HV.......................K......................Q....................................
CEMacaque    .S...HV.......................K......................Q....................................
OBaboon      .S...HV.......................K......................Q....................................
Marmoset     ...L..T.C..............RM..........................N...........................N..........
BSqMonkey    ......A.C..............RM..........................N...........................N..........

                    280       290       300       310       320       330       340       350       360
                      |         |         |         |         |         |         |         |         |
Human        QFADVIVLFGARLNWILHFGLPPRYQPDVKFIQVDICAEELGNNVKPAVTLLGNIHAVTKQLLEELDKTPWQYPPESKWWKTLREKMKSN
Chimpanzee   ...............................................T..........................................
Gorilla      ..........................................................................................
Orangutan    .......................................................N.................S................
Gibbon       .......................................................N..................................
IRMacaque    .....................................................D.N.........F........................
CRMacaque    .....................................................D.N.........F........................
CEMacaque    .....................................................D.N..................................
OBaboon      .....................................................D.N..................................
Marmoset     ...............................................T.....DVS..S..........S.E..................
BSqMonkey    .....................................................D.S..S..........S.V.........S........

                    370       380       390       400       410       420       430       440       450
                      |         |         |         |         |         |         |         |         |
Human        EAASKELASKKSLPMNYYTVFYHVQEQLPRDCFVVSEGANTMDIGRTVLQNYLPRHRLDAGTFGTMGVGLGFAIAAAVVAKDRSPGQWII
Chimpanzee   .......................I..................................................................
Gorilla      ..........................................................................................
Orangutan    ...................................................CR........................M.........RV.
Gibbon       .............................................................................M.........RV.
IRMacaque    .........RE..................................................................M.........RV.
CRMacaque    .........RE..................................................................M.........RV.
CEMacaque    .........RE..................................................................M.........RV.
OBaboon      .............................................................................M.........RV.
Marmoset     .............................................................................M.....H...RV.
BSqMonkey    .............................T...............................................M..........V.
```

```
                     460       470       480       490       500       510       520       530       540
                       |         |         |         |         |         |         |         |         |
Human        CVEGDSAFGFSGMEVETICRYNLPIILLVVNNNGIYQGFDTDTWKEMLKFQDATAVVPPMCLLPNSHYEQVMTAFGGKGYFVQTPEELQK
Chimpanzee   ..........................................................................................
Gorilla      ..........................................................................................
Orangutan    ............................I.....................RG......................................
Gibbon       ..................................................R.......................................
IRMacaque    .....................................................AS...........................K.......
CRMacaque    .....................................................AS...........................K.......
CEMacaque    .....................................................AS...................................
OBaboon      .....................................................AS...................................
Marmoset     ........................................M.A...T.....P.................I...........E.......
BSqMonkey    ........................................MN....T.....P...I.........................E.......

                     550       560       570
                       |         |         |
Human        SLRQSLADTTKPSLINIMIEPQATRKAQDFHWLTRSNM
Chimpanzee   ................V.....................
Gorilla      ......................................
Orangutan    ......................................
Gibbon       ......................................
IRMacaque    .........A................T...........
CRMacaque    .........A................T...........
CEMacaque    .........A................T...........
OBaboon      .........A................T...........
Marmoset     .........S............................
BSqMonkey    ...L..................................
```

ALDH3A2

```
                      10        20        30        40        50        60        70        80        90
                       |         |         |         |         |         |         |         |         |
Human        MELEVRRVRQAFLSGRSRPLRFRLQQLEALRRMVQEREKDILTAIAADLCKSEFNVYSQEVITVLGEIDFMLENLPEWVTAKPVKKNVLT
Chimpanzee   ..........................................A...S.............................EG....................
Gorilla      ..........................................A...................L............................
Orangutan    ..........................................A...............................................
Gibbon       ..R...........S...........................A...............................................
IRMacaque    ..R..Q....................................A..........L.A...............................L..
CRMacaque    ..R..Q....................................A..........L.A...............................L..
CEMacaque    ..R..Q....................................A..........L.A...............................L..
OBaboon      ..R..Q....................................A..........L.A...............................L..
Marmoset     ..H..Q....................................A..GS........A......A...............I........L..
BSqMonkey    ..R..Q....................................A..RS........A.......................A.......L..

                     100       110       120       130       140       150       160       170       180
                       |         |         |         |         |         |         |         |         |
Human        MLDEAYIQPQPLGVVLIIGAWNYPFVLTIQPLIGAIAAGNAVIIKPSELSENTAKILAKLLPQYLDQDLYIVINGGVEETTELLKQRFDH
Chimpanzee   .......................................................................I..................
Gorilla      .....................................................................................R....
Orangutan    ...................................V...................M..................................
Gibbon       ..........................................................................................
IRMacaque    .M.........................I............................V.............V...................
CRMacaque    .M.........................I............................V.............V...................
CEMacaque    .M.........................I............................V.............V...................
OBaboon      .M.........................I............................V.............V...................
Marmoset     ......L...............................................T...................................
BSqMonkey    ......L...............................................T...................................

                     190       200       210       220       230       240       250       260       270
                       |         |         |         |         |         |         |         |         |
Human        IFYTGNTAVGKIVMEAAAKHLTPVTLELGGKSPCYIDKDCDLDIVCRRITWGKYMNCGQTCIAPDYILCEASLQNQIVWKIKETVKEFYG
Chimpanzee   ............I.............................................................................
Gorilla      ..........................................................................................
Orangutan    ..........................................................................................
Gibbon       .......................................S..................................................
IRMacaque    ..........................................................................S...............
CRMacaque    ..........................................................................S...............
CEMacaque    ..........................................................................S...............
OBaboon      ..........................................................................S...............
Marmoset     .L.....T......................................................................Q...........
BSqMonkey    .L............................................................................Q...........

                     280       290       300       310       320       330       340       350       360
                       |         |         |         |         |         |         |         |         |
Human        ENIKESPDYERIINLRHFKRILSLLEGQKIAFGGETDEATRYIAPTVLTDVDPKTKVMQEEIFGPILPIVPVKNVDEAINFINEREKPLA
Chimpanzee   ..........................................................................................
Gorilla      ..........................................................................................
Orangutan    ..........................................................................................
Gibbon       ..........................................................................................
IRMacaque    ...............................L.................................V.............D..........
CRMacaque    ...............................L.................................V.............D..........
CEMacaque    ...............................L.................................V.............D..........
OBaboon      ...............................L.................................V.............D..........
Marmoset     ..V...........H..................................................V........................
BSqMonkey    ..V..............................................................V........................

                     370       380       390       400       410       420       430       440       450
                       |         |         |         |         |         |         |         |         |
Human        LYVFSHNHKLIKRMIDETSSGGVTGNDVIMHFTLNSFPFGGVGSSGMGAYHGKHSFDTFSHQRPCLLKSLKREGANKLRYPPNSQSKVDW
Chimpanzee   ................................M.........................................................
Gorilla      ..........................................................................................
Orangutan    ................................M.........................................................
Gibbon       ..........................................................................................
IRMacaque    ..........................................................................................
CRMacaque    ..........................................................................................
CEMacaque    ..........................................................................................
OBaboon      ..........................................................................................
Marmoset     ................................M.....................T.................................Y.
BSqMonkey    .......N........................M.........................................................
```

```
                    460       470       480       490       500
                      |         |         |         |         |
Human        GKFFLLKRFNKEKLGLLLLTFLGIVAAVLVKKYQAVLRRKALLIFLVVHRLRWSSKQR
Chimpanzee   ..........................................................
Gorilla      ..........................................................
Orangutan    ..........................................................
Gibbon       ..........................................................
IRMacaque    ......R..........VF.......................................
CRMacaque    ......R..........V........................................
CEMacaque    ......R..........V........................................
OBaboon      ..................F.......................................
Marmoset     ..............................................I...........
BSqMonkey    ...........Q.....F....................................T...
```

Green font highlighted in yellow: Human and Neandertal amino acid residue differ from chimpanzee.

SLC27A2

```
                    10        20        30        40        50        60        70        80        90
                     |         |         |         |         |         |         |         |         |
Human        MLSAIYTVLAGLLFLPLLVNLCCPYFFQDIGYFLKVAAVGRRVRSYGKRRPARTILRAFLEKARQTPHKPFLLFRDETLTYAQVDRRSNQ
Chimpanzee   ..........................L...............................................................
Gorilla      ....................................V.....................................................
Orangutan    ..........................................................................................
Gibbon       ....................F..........................R.......V........R.........................
IRMacaque    .............................V............A....Q.....................................Q....
CRMacaque    .............................V............A....Q.....................................Q....
CEMacaque    .............................V............A....Q.....................................Q....
OBaboon      .............................V............A....Q.................................S...Q....
Marmoset     ..P...........................S...H..S.A..T....Q...V..V...................................
BSqMonkey    ..P..........................MS...Q..S.A.......Q...V.....E................................

                   100       110       120       130       140       150       160       170       180
                     |         |         |         |         |         |         |         |         |
Human        VARALHDHLGLRQGDCVALLMGNEPAYVWLWLGLVKLGCAMACLNYNIRAKSLLHCFQCCGAKVLLVSPELQAAVEEILPSLKKDDVSIY
Chimpanzee   ...................F..............................................A.......................
Gorilla      ...................F..............................................A.......................
Orangutan    ...................F..............................................A.......................
Gibbon       ...................F..............................................A.......................
IRMacaque    ...................F..............................................A..D....................
CRMacaque    ...................F..............................................A..D....................
CEMacaque    ...................F..............................................A..D....................
OBaboon      ...................F..............................................A..D....................
Marmoset     ...................F.........................C........R...........A..................-....
BSqMonkey    ...................F.........................C....................A...............Q..-....

                   190       200       210       220       230       240       250       260       270
                     |         |         |         |         |         |         |         |         |
Human        YVSRTSNTDGIDSFLDKVDEVSTEPIPESWRSEVTFSTPALYIYTSGTTGLPKAAMITHQRIWYGTGLTFVSGLKADDVIYITLPFYHSA
Chimpanzee   ..........................................................................................
Gorilla      ......................................T...................................................
Orangutan    ..........................................................................................
Gibbon       ..........................................................................................
IRMacaque    ..........V...........A..T...........................................................L....
CRMacaque    ..........V...........A..T...........................................................L....
CEMacaque    ..........V...........A..T...........................................................L....
OBaboon      ..........V...........A..T...........................................................L....
Marmoset     ..........VH.......................V.N...L.F.....S...........V.......................L....
BSqMonkey    ..........VH.......................V.N.H.........S...........V.......................L....

                   280       290       300       310       320       330       340       350       360
                     |         |         |         |         |         |         |         |         |
Human        ALLIGIHGCIVAGATLALRTKFSASQFWDDCRKYNVTVIQYIGELLRYLCNSPQKPNDRDHKVRLALGNGLRGDVWRQFVKRFGDICIYE
Chimpanzee   .............................................................T............................
Gorilla      ..........................................................................................
Orangutan    ..........................................................................................
Gibbon       ......................................................Q.................E.................
IRMacaque    .....F.............................................T......................................
CRMacaque    .....F.............................................T......................................
CEMacaque    .....F.............................................T......................................
OBaboon      .....F.............................................T.................................V....
Marmoset     ..M............V...S...............................L........................K.........H...
BSqMonkey    ..M................S......................................H.......V.........K.........H...

                   370       380       390       400       410       420       430       440       450
                     |         |         |         |         |         |         |         |         |
Human        FYAATEGNIGFMNYARKVGAVGRVNYLQKKIITYDLIKYDVEKDEPVRDENGYCVRVPKGEVGLLVCKITQLTPFNGYAGAKAQTEKKKL
Chimpanzee   ..........................................................................................
Gorilla      ......................................................I...................................
Orangutan    ......................................................................K...................
Gibbon       ..........................................................................................
IRMacaque    ..............T............................................................S..............
CRMacaque    ..............T............................................................S..............
CEMacaque    ..............T............................................................S..............
OBaboon      ..............T..................................G.....I....................S..............
Marmoset     ...........L..T.....I.........A...............I.......I....................S....G.........
BSqMonkey    ..........LL..T...............V.S......................K...................S....G.........
```

```
                    460       470       480       490       500       510       520       530       540
                      |         |         |         |         |         |         |         |         |
Human        RDVFKKGDLYFNSGDLLMVDHENFIYFHDRVGDTFRWKGENVATTEVADTVGLVDFVQEVNVYGVHVPDHEGRIGMASIKMKENHEFDGK
Chimpanzee   ..........................................................................................
Gorilla      ..........................................................................................
Orangutan    ..........................................................................................
Gibbon       ....................D............................I........................................
IRMacaque    ....................R............................I........................................
CRMacaque    ....................R............................I........................................
CEMacaque    ....................R............................I........................................
OBaboon      ..................I.R............................I........................................
Marmoset     ....................R............................II..L..............G.....................
BSqMonkey    ....................R............................II..L..............G.....................

                    550       560       570       580       590       600       610       620
                      |         |         |         |         |         |         |         |
Human        KLFQHIADYLPSYARPRFLRIQDTIEITGTFKHRKMTLVEEGFNPAVIKDALYFLDDTAKMYVPMTEDIYNAISAKTLKL
Chimpanzee   ................................................................................
Gorilla      ................................................................................
Orangutan    ...................................V............................................
Gibbon       ...........T.......................V..........I.................................
IRMacaque    ...................................V........................T...................
CRMacaque    ...................................V........................T...................
CEMacaque    ...................................V........................T...................
OBaboon      ...................................V........................T...................
Marmoset     ....................M..............V..................................K....NS...
BSqMonkey    .F..................M............C.V.........S..............S...........V..NS...
```

<u>**Green font highlighted in yellow:**</u> Human and Neandertal amino acid residue differ from chimpanzee.

```
ASCL1                  10        20        30        40        50        60        70        80        90
                        |         |         |         |         |         |         |         |         |
Human        MQAHELFRYFRMPELVDFRQYVRTLPTNTLMGFGAFAALTTFWYATRPKPLKPPCDLSMQSVEVAGSGGARRSALLDSDEPLVYFYDDVT
Chimpanzee   ..........................................................................................
Gorilla      ..........................................................................................
Orangutan    ..........................................................................................
Gibbon       .................L........................................................................
IRMacaque    .................................................A....................................E...
CRMacaque    .................................................A....................................E...
CEMacaque    .................................................A....................................E...
OBaboon      .................................................A....................................E...
Marmoset     ................................................RA.......................T.........C..E...
BSqMonkey    .................................................A.......................T.........C..E...

                      100       110       120       130       140       150       160       170       180
                        |         |         |         |         |         |         |         |         |
Human        TLYEGFQRGIQVSNNGPCLGSRKPDQPYEWLSYKQVAELSECIGSALIQKGFKTAPDQFIGIFAQNRPEWVIIEQGCFAYSMVIVPLYDT
Chimpanzee   ......................................................T...................................
Gorilla      ..........................................................................................
Orangutan    ...................................................................................V......
Gibbon       ..........................................................................................
IRMacaque    ......................................M...L.........TAT...................................
CRMacaque    ......................................M...L.........TAT...................................
CEMacaque    ......................................M...L.........TAT...................................
OBaboon      ......................................M...L.........TAT...................................
Marmoset     ..........H....................................L.....A.....V..............................
BSqMonkey    ..........H....................................L.....AT...CV..............................

                      190       200       210       220       230       240       250       260       270
                        |         |         |         |         |         |         |         |         |
Human        LGNEAITYIVNKAELSLVFVDKPEKAKLLLEGVENKLIPGLKIIVVMDAYGSELVERGQRCGVEVTSMKAMEDLGRANRRKPKPPAPEDL
Chimpanzee   ..........................................................................................
Gorilla      ................................................................................N.........
Orangutan    ..........................I..................L.............K..............................
Gibbon       .............................................L.............K....II........................
IRMacaque    .....V...............................T.S.....L.............K....II.L......................
CRMacaque    .....V...............................T.S.....L.............K....II.L......................
CEMacaque    .....V...............................T.S.....L.............K....II.L......................
OBaboon      .....V....................E..........T.S.....L.............K....II.L........V.............
Marmoset     ..S......L...........................T.......L............KK.....I.............Q..........
BSqMonkey    ..S..........................................L............KK.....I..........T..Q..........

                      280       290       300       310       320       330       340       350       360
                        |         |         |         |         |         |         |         |         |
Human        AVICFTSGTTGNPKGAMVTHRNIVSDCSAFVKATENTVNPCPDDTLISFLPLAHMFERVVECVMLCHGAKIGFFQGDIRLLMDDLKVLQP
Chimpanzee   ..........................................................................................
Gorilla      ..........................................................................................
Orangutan    ..........................................................................................
Gibbon       .................I........................................................................
IRMacaque    ..V.............LI..................S.....................................................
CRMacaque    ..V.............LI..................S.....................................................
CEMacaque    ..V.............LI..................S.....................................................
OBaboon      ..V.............LI..................S.....................................................
Marmoset     ..V.............LI....V.........M.....I.R...I.............................................
BSqMonkey    ................LI....V.........M.....I.....I.............................................

                      370       380       390       400       410       420       430       440       450
                        |         |         |         |         |         |         |         |         |
Human        TVFPVVPRLLNRMFDRIFGQANTTLKRWLLDFASKRKEAELRSGIIRNNSLWDRLIFHKVQSSLGGRVRLMVTGAAPVSATVLTFLRAAL
Chimpanzee   ..........................................................................................
Gorilla      ..........................................................................................
Orangutan    .I........................................................................................
Gibbon       .I........................................................................................
IRMacaque    ..................................................................K.......................
CRMacaque    ..................................................................K.......................
CEMacaque    ..................................................................K.......................
OBaboon      ..........................................................................................
Marmoset     .....................................................K...R.I..............................
BSqMonkey    .............................................V.......K.....I...........I..................
```

```
                    460       470       480       490       500       510       520       530       540
                      |         |         |         |         |         |         |         |         |
Human        GCQFYEGYGQTECTAGCCLTMPGDWTAGHVGAPMPCNLIKLVDVEEMNYMAAEGEGEVCVKGPNVFQGYLKDPAKTAEALDKDGWLHTGD
Chimpanzee   .............A............................................................................
Gorilla      ..........................................................................................
Orangutan    ..........................................................................................
Gibbon       ....................................S....................................V................
IRMacaque    ...................S.....................................................V................
CRMacaque    ...................S.....................................................V................
CEMacaque    ...................S.....................................................V................
OBaboon      ...................S......................................................................
Marmoset     .................................................I..K.....................................
BSqMonkey    ....................V................W..............K..................E..................

                    550       560       570       580       590       600       610       620       630
                      |         |         |         |         |         |         |         |         |
Human        IGKWLPNGTLKIIDRKKHIFKLAQGEYIAPEKIENIYMRSEPVAQVFVHGESLQAFLIAIVVPDVETLCSWAQKRGFEGSFEELCRNKDV
Chimpanzee   .....................................V....................................................
Gorilla      .....................................V....................................................
Orangutan    .....................................V......H........H..................R.................
Gibbon       .....................................V..................................R.................
IRMacaque    .....................................I...AI.........................RP.......D............
CRMacaque    .....................................I...AI.........................RP.......D............
CEMacaque    .....................................I...AI.........................RP.......D............
OBaboon      .....................................I...AI.........................RP.......D............
Marmoset     .....................................L..........................I.......R...LD............
BSqMonkey    .....................................L..........................S.I.....R....D............

                    640       650       660       670       680       690
                      |         |         |         |         |         |
Human        KKAILEDMVRLGKDSGLKPFEQVKGITLHPELFSIDNGLLTPTMKAKRPELRNYFRSQIDDLYSTIKV
Chimpanzee   ..................................................M.........E.......
Gorilla      ............................................................E.......
Orangutan    ............................................................E.......
Gibbon       ............................................................E.......
IRMacaque    ..........................A.................................E.......
CRMacaque    ..........................A.................................E.......
CEMacaque    ..........................A.................................E.......
OBaboon      ..........................A.................................E.......
Marmoset     ..........................A.................................E.......
BSqMonkey    ..........................A.......VY........................E....V..
```

```
AMACR                10        20        30        40        50        60        70        80        90
                      |         |         |         |         |         |         |         |         |
Human        MALQGISVVELSGLAPGPFCAMVLADFGARVVRVDRPGSRYDVSRLGRGKRSLVLDLKQPRGAAVLRRLCKRSDVLLEPFRRGVMEKLQL
Chimpanzee   ..........................................................................................
Bonobo       ..........................................................................................
Orangutan    .....................K................................................E...................
Siamang      ......................................F...............................E...................
Gibbon       ......................................F...............................E..........G........
IRMacaque    ......L.L..A......................E....H.............A................A..........S........
CRMacaque    ......L.L..A......................E....H.............A................A..........S........
CEMacaque    ......L.L..A......................E....H.............A................A..........S........
LTMacaque    ......L.L..A......................E....H.............A................A..........S........
OBaboon      ......L....A......................E....H.............A................A..........S........
DLangur      ...R.......A......................E....H..M...........................A..........S........
Marmoset     ...........A........G..........I..........L.G.A......A........V.......AK.........S....R...
BSqMonkey    .T.........A........G..........M............G.A......A........V.......AQ.........S....R...

                    100       110       120       130       140       150       160       170       180
                      |         |         |         |         |         |         |         |         |
Human        GPEILQRENPRLIYARLSGFGQSGSFCRLAGHDINYLALSGVLSKIGRSGENPYAPLNLLADFAGGGLMCALGIIMALFDRTRTGKGQVI
Chimpanzee   ..........................SW.......................................V......................
Bonobo       ..........................SW.......................................V......................
Orangutan    ..........................S...............................................................
Siamang      ..........................S....................................................E...S......
Gibbon       ..........................S....................................................E...S......
IRMacaque    .......D.........T........S.....................N.....................V...M....E...S......
CRMacaque    .......D.........T........S.....................N.....................V...M....E...S......
CEMacaque    .......D..K......T........S.....................N.....................V...M....E...S......
LTMacaque    .......D..K......T........S.....................N.....................V...M....E...S......
OBaboon      .......D.........T........S.....................N.....................V...M....E...S......
DLangur      .......D.........T........SQ....................N.....................VF..M....E...S......
Marmoset     ..........................S......................D.............................E...S......
BSqMonkey    ..........................S......................D.............................E...S......

                    190       200       210       220       230       240       250       260       270
                      |         |         |         |         |         |         |         |         |
Human        DANMVEGTAYLSSFLWKTQKLSLWEAPRGQNMLDGGAPFYTTYRTADGEFMAVGAIEPQFYELLIKGLGLKSDELPNQMSMDDWPEMKKK
Chimpanzee   ....................S...................M.................................................
Bonobo       ....................S...................M.................................................
Orangutan    .......A............S.....................................................................
Siamang      ..S....A..................................................................................
Gibbon       ..S....A..................S...............................................................
IRMacaque    ....................S..........I..........................................................
CRMacaque    ....................S..........I..........................................................
CEMacaque    ....................S..........I..........................................................
LTMacaque    ....................S..........I..........................................................
OBaboon      ....................S..........I..........................................................
DLangur      ....................S.....................................................................
Marmoset     .....................N..K...........................................E..P.........N........
BSqMonkey    ....................FN.................................................P.........N........

                    280       290       300       310       320       330       340       350       360
                      |         |         |         |         |         |         |         |         |
Human        FADVFAEKTKAEWCQIFDGTDACVTPVLTFEEVVHHDHNKERGSFITSEEQDVSPRPAPLLLNTPAIPSFKRDPFIGEHTEEILEEFGFS
Chimpanzee   ......K........................................N...........................V..............
Bonobo       ......K........................................N...........................V..............
Orangutan    ......K............A.........L........H....................................V..............
Siamang      ......K........................................N....L......................V..............
Gibbon       ......K........................................N....L......................V..............
IRMacaque    ..A...K......................L........I........N...SM........S.............V........D.....
CRMacaque    ..A...K......................L........I........N...SM........S.............V........D.....
CEMacaque    ..A...K......................L........I........N...SM........S.............V........D.....
LTMacaque    ..A...K......................L........S........N...SI........S.............V........D.....
OBaboon      ..A...K......................L........S........N...SM........S.............V........D.....
DLangur      ..A...K......................L........S........N...NM........S.............V........D.....
Marmoset     ......K.............N.............S...............R..........SD...V........V........K.....
BSqMonkey    ......K...........................S............N.............SD...V........V........K.....
```

```
                       370       380
                         |         |
Human        REEIYQLNSDKIIESNKVKASL
Chimpanzee   ......................
Bonobo       ......................
Orangutan    ......................
Siamang      ......................
Gibbon       .......Y..............
IRMacaque    ....D..K..............
CRMacaque    ....D..K..............
CEMacaque    ....D..K..............
LTMacaque    ....D..K...V..........
OBaboon      ....D..K..............
DLangur      ....D..K..............
Marmoset     LK..D..T.......H....G.
BSqMonkey    LK..D..T.......H......
```

Red font highlighted in yellow: Human amino acid residue differs from Neandertal and chimpanzee. Note that there is a human cSNP encoding a Ser at this position whereby both the human and Neandertal amino acid residue differs from chimpanzee.

Green font highlighted in yellow: Human and Neandertal amino acid residue differ from chimpanzee.

ACOX2

```
                     10        20        30        40        50        60        70        80        90
                      |         |         |         |         |         |         |         |         |
Human        MGSPVHRVSLGDTWSRQMHPDIESERYMQSFDVERLTNILDGGAQNTALRRKVESIIHSYPEFSCKDNYFMTQNERYKAAMRRAFHIRLI
Chimpanzee   .................................................................................Q........
Gorilla      ..........................................................................................
Orangutan    ..........................................................................................
Gibbon       ..................................Q..........................................E............
IRMacaque    .....................M..........................................R.........................
CRMacaque    .....................M..........................................R.........................
CEMacque     .....................M..........................................R.........................
OBaboon      ...A.................M..........................................R.........................
Marmoset     ..........................H.....................................R..D.....................T
BSqMonkey    ..........................HI...................E................R..D......................

                    100       110       120       130       140       150       160       170       180
                      |         |         |         |         |         |         |         |         |
Human        ARRLGWLEDGRELGYAYRALSGDVALNIHRVFVRALRSLGSEEQIAKWDPLCKNIQIIATYAQTELGHGTYLQGLETEATYDAATQEFVI
Chimpanzee   ..........................................................................................
Gorilla      ..........................................................T...............................
Orangutan    ..H.............................LK.......D................................................
Gibbon       ................................L.........................................................
IRMacaque    .Q..............................LK........................................................
CRMacaque    .Q..............................LK........................................................
CEMacque     .Q..............................LK........................................................
OBaboon      .Q..............................LK........................................................
Marmoset     .Q.....A..P........I.............K......T.................T...............................
BSqMonkey    .Q.....A.....................G..QK......T.................................................

                    190       200       210       220       230       240       250       260       270
                      |         |         |         |         |         |         |         |         |
Human        HSPTLTATKWWPGDLGRSATHALVQAQLICSGARRGMHAFIVPIRSLQDHTPLPGIIIGDIGPKMDFDQTDNGFLQLNHVRVPRENMLSR
Chimpanzee   ..................................Q.......................................................
Gorilla      .........................................................T................................
Orangutan    .........................................................V................................
Gibbon       .........................................................V................................
IRMacaque    ............................V..................R.....................M.....R..............
CRMacaque    ............................V..................R.....................M.....R..............
CEMacque     ............................V..................R.....................M.....R..............
OBaboon      ............................V..................R.........V...........M........Y...........
Marmoset     .........................................................V................................
BSqMonkey    ..................................................A.....TV................................

                    280       290       300       310       320       330       340       350       360
                      |         |         |         |         |         |         |         |         |
Human        FAQVLPDGTYVKLGTAQSNYLPMVVVRVELLSGEILPILQKACVIAMRYSVIRRQSRLRPSDPEAKVLDYQTQQQKLFPQLAISYAFHFL
Chimpanzee   ..........................................................................................
Gorilla      ..........................................................................................
Orangutan    ..................................V...........T...........................................
Gibbon       ...............T..........................................................................
IRMacaque    ..........I....................L..V..T........T.........Q.................................
CRMacaque    ..........I....................L..V..T........T.........Q.................................
CEMacque     ..........I....................L..V..T........T.........Q.................................
OBaboon      ..........I....................L..V..T........T.........Q.................................
Marmoset     ...............................L.....L..................Q.....Q...........................
BSqMonkey    ...............................L.....L........T.........Q.....Q...........................

                    370       380       390       400       410       420       430       440       450
                      |         |         |         |         |         |         |         |         |
Human        AVSLLEFFQHSYTAILNQDFSFLPELHALSTGMKAMMSEFCTQGAEMCRRACGGHGYSKLSGLPSLVTKLSASCTYEGENTVLYLQVARF
Chimpanzee   ......................................................................L...................
Gorilla      ......................................................................L...................
Orangutan    .................R........................................................................
Gibbon       .................R.......................I................................................
IRMacaque    ..........A......R...L....................................................................
CRMacaque    ..........A......R...L....................................................................
CEMacque     ..........A......R...L....................................................................
OBaboon      ..........A......R...L....................................................................
Marmoset     .........R..S....R...I................D..............................V....................
BSqMonkey    .........R..S....R..HI................D..............................V....................
```

```
                     460       470       480       490       500       510       520       530       540
                       |         |         |         |         |         |         |         |         |
Human         LVKSYLQTQMSPGSTPQRSLSPSVAYLTAPDLARCPAQRAADFLCPELYTTAWAHVAVRLIKDSVQHLQTLTQSGADQHEAWNQTTVIHL
Chimpanzee    ....................P.....................................................................
Gorilla       ....................P.....................................................................
Orangutan     ....................P....................................A.............M..................
Gibbon        ....................P........A................K..........A................................
IRMacaque     ....................P......AT............................A.......H........................
CRMacaque     ....................P......AT............................A.......H........................
CEMacque      ....................P......AT............................A.......H........................
OBaboon       ....................P......AT............................A.......H........................
Marmoset      ...N.......A..AQ....P.........V..........................A.......H........................
BSqMonkey     ...N..........A.....P.........V...............K..........A.......H........................

                     550       560       570       580       590       600       610       620       630
                       |         |         |         |         |         |         |         |         |
Human         QAAKVHCYYVTVKGFTEALEKLENEPAIQQVLKRLCDLHAIHGILTNSGDFLHDAFLSGAQVDMARTAYLDLLRLIRKDAILLTDAFDFT
Chimpanzee    .........................................................................C................
Gorilla       ....A....................................................................H................
Orangutan     ....A......................................................D..............................
Gibbon        ....A....I......................Q.........................................Q...............
IRMacaque     ....A....................S................................................................
CRMacaque     ....A....................S................................................................
CEMacque      ....A....................S................................................................
OBaboon       ....A....................S...............................................C................
Marmoset      ....A..........A......................................S..................F................
BSqMonkey     ....A..........A......................................S..................C................

                     640       650       660       670       680
                       |         |         |         |         |
Human         DQCLNSALGCYDGNVYERLFQWAQKSPTNTQENPAYEEYIRPLLQSWRSKL
Chimpanzee    ...................................................
Gorilla       ...................................................
Orangutan     ...............................................K...
Gibbon        ...................................................
IRMacaque     .E.................................................
CRMacaque     .E.................................................
CEMacque      .E.................................................
OBaboon       .E.................................................
Marmoset      ........................R..........................
BSqMonkey     ...................................................
```

Green font highlighted in yellow: Human and Neandertal amino acid residue differ from chimpanzee.

```
ACOX3
                    10        20        30        40        50        60        70        80        90
                     |         |         |         |         |         |         |         |         |
Human       MASTVEGGDTALLPEFPRGPLDAYRARASFSWKELALFTEGEGMLRFKKTIFSALENDPLFARSPGADLSLEKYRELNFLRCKRIFEYDF
Chimpanzee  ..........................................D...............................................
Gorilla     .....................N....................DV..............................................
Orangutan   ..........................................D.......................................R.......
IRMacaque   ..P......A................................D............................D..................
CRMacaque   ..P......A................................D............................D..................
CEMacaque   ..P......A................................D............................D..................
OBaboon     ..P......A................................D............................DR.................
Marmoset    ..P.LK...A............S...................D........................H.........S............
BSqMonkey   ..P....R.AT.F.........T...................DV............I..........H......................

                   100       110       120       130       140       150       160       170       180
                     |         |         |         |         |         |         |         |         |
Human       LSVEDMFKSPLKVPALIQCLGMYDSSLAAKYLLHSLVFGSAVYSSGSERHLTYIQKIFRMEIFGCFALTELSHGSNTKAIRTTAHYDPAT
Chimpanzee  ..........................................................................................
Gorilla     .......Q......T...........................................................................
Orangutan   .............LT.................I........I................................................
IRMacaque   ..................................T.......................S...............................
CRMacaque   ..................................T.......................S...............................
CEMacaque   ..................................T.......................S...............................
OBaboon     ..................................T.......................S...............................
Marmoset    ..AG.........ST.........W......V..T.I.....F.......F.......S...........................N...
BSqMonkey   ..AG.V.E.....ST.........W.........T.I...V.V.A.....FA..P...S...........................N...

                   190       200       210       220       230       240       250       260       270
                     |         |         |         |         |         |         |         |         |
Human       EEFIIHSPDFEAAKFWVGNMGKTATHAVVFAKLCVPGDQCHGLHPFIVQIRDPKTLLPMPGVMVGDIGKKLGQNGLDNGFAMFHKVRVPR
Chimpanzee  ..........................................................................................
Gorilla     .................................Y........................................................
Orangutan   .................................Y........................................................
IRMacaque   K................................H................................M.......................
CRMacaque   K................................H................................M.......................
CEMacaque   K................................H................................M.......................
OBaboon     K................................H................................M.......................
Marmoset    ...............................Q.HM..G.........I.................................V........
BSqMonkey   ...............................Q.H...G.........I........F........................V........

                   280       290       300       310       320       330       340       350       360
                     |         |         |         |         |         |         |         |         |
Human       QSLLNRMGDVTPEGTYVSPFKDVRQRFGASLGSLSSGRVSIVSLAILNLKLAVAIALRFSATRRQFGPTEEEEIPVLEYPMQQWRLLPYL
Chimpanzee  ............................V.............................................................
Gorilla     ............K.............................................................................
Orangutan   ..................S......H...T.....WS.....................................................
IRMacaque   ............................V...G........L...VV.....I................Q....A........C......
CRMacaque   ............................V...G........L...VV.....I................Q....A........C......
CEMacaque   ............................V...G........L...VV.....I................Q....A........C......
OBaboon     ............................V...G........L...VV.....I................Q....A........C......
Marmoset    HN....................A............W...V.MAM.VT......S..A......................QT.........
BSqMonkey   ......................A............W...T.MGM.VT.....LS..V.............D........QT.........

                   370       380       390       400       410       420       430       440       450
                     |         |         |         |         |         |         |         |         |
Human       AAVYALDHFSKSLFLDLVELQRGLASGDRSARQAELGREIHALASASKPLASWTTQQGIQECREACGGHGYLAMNRLGVLRDDNDPNCTY
Chimpanzee  ..........................................................................................
Gorilla     ..........................................................................................
Orangutan   ..I..............M...Q....................................................................
IRMacaque   ..A.T..................R......................G.......A...................................
CRMacaque   ..A....................R......................G.......A...................................
CEMacaque   ..A....................R......................G.......A...................................
OBaboon     ..A....................R....C.................G.......A...................................
Marmoset    ..A.......T.....M........A....................G.......A..................V................
BSqMonkey   ..A..........V..........A.....................G.......A..................V................
```

```
                    460       470       480       490       500       510       520       530       540
                      |         |         |         |         |         |         |         |         |
Human        EGDNNILLQQTSNYLLGLLAHQVHDGACFRSPLKSVDFLDAYPGILDQKFEVSSVADCLDSAVALAAYKWLVCYLLRETYQKLNQEKRSG
Chimpanzee   .......................Q..................................................................
Gorilla      .......................Q..................................................................
Orangutan    .......................Q..................................................................
IRMacaque    .....V...............R.R...................S..........I...................................
CRMacaque    .....V...............R.R...................S..............................................
CEMacaque    .....V...............R.R...................S..............................................
OBaboon      .....V...............R.Q...................S..............................................
Marmoset     .....V..............LR.R...RI...........S..S....R..............................HE.........
BSqMonkey    .....V..............CR.Q................S.......R..............................HE.........

                    550       560       570       580       590       600       610       620       630
                      |         |         |         |         |         |         |         |         |
Human        SSDFEARNKCQVSHGRPLALAFVELTVVQRFHEHVHQPSVPPSLRAVLGRLSALYALWSLSRHAALLYRGGYFSGEQAGEVLESAVLALC
Chimpanzee   ........N.............................C........................T..........................
Gorilla      ......................................C...................................................
Orangutan    ......................................C...................................................
IRMacaque    ........N.............L...............C...................................................
CRMacaque    ........N.............L...............C......................H............................
CEMacaque    ........N.............L...............C...................................................
OBaboon      ........N.............L...............C...................................................
Marmoset     .G......N...F.........M..M.L.S......R.C.....................N..T..........D....R.....I.T..
BSqMonkey    .D......N...F.........M....L..........C.....................NC.................R.....I.T..

                    640       650       660       670       680       690       700
                      |         |         |         |         |         |         |
Human        SQLKDDAVALVDVIAPPDFVLDSPIGRADGELYKNLWGAVLQESKVLERASWWPEFSVNKPVIGSLKSKL
Chimpanzee   ......................................................................
Gorilla      ......................................................................
Orangutan    ...................I...................I.....A........................
IRMacaque    ............................................N.........................
CRMacaque    ......................................................................
CEMacaque    ......................................................................
OBaboon      ......................................................................
Marmoset     .......................................I..D.Q....................V....
BSqMonkey    ...................I......................D...................T.......
```

Green font highlighted in yellow: Human and Neandertal amino acid residue differ from chimpanzee.

EHHADH

```
                     10        20        30        40        50        60        70        80        90
                      |         |         |         |         |         |         |         |         |
Human        MAEYTRLHNALALIRLRNPPVNAISTTLLRDIKEGLQKAVIDHTIKAIVICGAEGKFSAGADIRGFSAPRTFGLTLGHVVDEIQRNEKPV
Chimpanzee   ..........................................................................................
Gorilla      .........................M................................................................
Orangutan    ..........................A....................................H.........F................
Gibbon       ..........................A....................................H.........F...N............
IRMacaque    ..........................AV...V...............................H......................V...
CRMacaque    ..........................AV...V...............................H......................V...
CEMacaque    ..........................AV...V...............................H......................V...
OBaboon      ..........................AV...V...............................H......................V...
Marmoset     ......-........F......V..MA...EME.......T......................H..........S..S........V...
BSqMonkey    ...............F.........MA...E.E.......T......................H.......L..S..S........V...

                    100       110       120       130       140       150       160       170       180
                      |         |         |         |         |         |         |         |         |
Human        VAAIQGMAFGGGLELALGCHYRIAHAEAQVGLPEVTLGLLPGARGTQLLPRLTGVPAALDLITSGRRILADEALKLGILDKVVNSDPVEE
Chimpanzee   .........................................................S................................
Gorilla      ..........................................................................................
Orangutan    .........................S..........................I.............H.......................
Gibbon       ...........A........................................I.............H.......................
IRMacaque    .........................T..........................I.............H...G...................
CRMacaque    .........................T..........................I.............H...G...................
CEMacaque    .........................T..........................I.............H...G...................
OBaboon      .................S.......T.........K................I.............H...G...E...............
Marmoset     ........L...................R..........V............I..........T..H...G...............LL..
BSqMonkey    ........L..............................V............I..........T..Y...G................L..

                    190       200       210       220       230       240       250       260       270
                      |         |         |         |         |         |         |         |         |
Human        AIRFAQRVSDQPLESRRLCNKPIQSLPNMDSIFSEALLKMRRQHPGCLAQEACVRAVQAAVQYPYEVGIKKEEELFLYLLQSGQARALQY
Chimpanzee   ..........................................................................................
Gorilla      ..........................................................................................
Orangutan    ..............................T...................A.................V..........F..........
Gibbon       ..............................T...........................................................
IRMacaque    ......T.......................T....V....QK.....................................F..........
CRMacaque    ......T.......................T....V....QK.....................................F..........
CEMacaque    ......T.......................T....V....QK.....................................F..........
OBaboon      ..............................T....V....QK.....................................F..........
Marmoset     ..................FK.............G.......K..........................S.......Q.............
BSqMonkey    ..................F....P......T..........K..........................S.......Q.............

                    280       290       300       310       320       330       340       350       360
                      |         |         |         |         |         |         |         |         |
Human        AFFAERKANKWSTPSGASWKTASARPVSSVGVVGLGTMGRGIVISFARARIPVIAVDSDKNQLATANKMITSVLEKEASKMQQSGHPWSG
Chimpanzee   ..........................................................................................
Gorilla      ..........................................................................................
Orangutan    ..L.......................................................................................
Gibbon       ..L.......................................................................................
IRMacaque    ........................Q...........................................I.....................
CRMacaque    ........................Q...........................................I.....................
CEMacaque    ........................Q...........................................I.....................
OBaboon      ........................Q...........................................I.....................
Marmoset     ..L.........S...........Q...........................................I.....................
BSqMonkey    ..L.........S...........Q.I......................G..................I.................A...

                    370       380       390       400       410       420       430       440       450
                      |         |         |         |         |         |         |         |         |
Human        PKPRLTSSVKELGGVDLVIEAVFEEMSLKKQVFAELSAVCKPEAFLCTNTSALDVDEIASSTDRPHLVIGTHFFSPAHVMKLLEVIPSQY
Chimpanzee   ........M.................................................................................
Gorilla      ........M.............................................I...................................
Orangutan    ........M.............................I...................................................
Gibbon       ........M...........................................................V...................R.
IRMacaque    ...K....M...............................................................................R.
CRMacaque    ...K....M...............................................................................R.
CEMacaque    ...K....M...............................................................................R.
OBaboon      ...K....M........................S......................................................R.
Marmoset     ........M...............D..........................S..........N...............I...........
BSqMonkey    ........M...............D................................L....S...............I.........R.
```

```
                    460       470       480       490       500       510       520       530       540
                      |         |         |         |         |         |         |         |         |
Human        SSPTTIATVMNLSKKIKKIGVVVGNCFGFVGNRMLNPYYNQAYFLLEEGSKPEEVDQVLEEFGFKMGPFRVSDLAGLDVGWKSRKGQGLT
Chimpanzee   ..........................................................................................
Gorilla      ..........................................................................................
Orangutan    ..........................................................................................
Gibbon       .......................................................................................D..
IRMacaque    .........................................T................................................
CRMacaque    .........................................T................................................
CEMacaque    .........................................T................................................
OBaboon      ......V..................................T................................................
Marmoset     .......................................S.T..............R.................................
BSqMonkey    .........................................T................................................

                    550       560       570       580       590       600       610       620       630
                      |         |         |         |         |         |         |         |         |
Human        GPTLLPGTPARKRGNRRYCPIPDVLCELGRFGQKTGKGWYQYDKPLGRIHKPDPWLSKFLSRYRKTHHIEPRTISQDEILERCLYSLINE
Chimpanzee   ....P........................................................Q..E.........................
Gorilla      ....P........................................................Q..E.........................
Orangutan    ....P........................................................Q..E.........................
Gibbon       ....P........................................................Q..E.........................
IRMacaque    ....P..................M.....................................Q..E.Y.......................
CRMacaque    ....P..................M.....................................Q..E.Y.......................
CEMacaque    ....P..................M.....................................Q..E.Y.......................
OBaboon      ....P..................M.....................................Q..E.Y.......................
Marmoset     ....P.........S..............................................Q..E.......I..R..............
BSqMonkey    ....P....................................................T...Q..E.......I..R..............

                    640       650       660       670       680       690       700       710       720
                      |         |         |         |         |         |         |         |         |
Human        AFRILGEGIAASPEHIDVVYLHGYGWPRHKGGPMFYASTVGLPTVLEKLQKYYRQNPDIPQLEPSDYLKKLASQGNPPLKEWQSLAGSPS
Chimpanzee   ..........................................................................................
Gorilla      ...........................................R..............................................
Orangutan    ..........................................................................................
Gibbon       ..........................................................................................
IRMacaque    ..H.......T...............................................................................
CRMacaque    ..H.......T...............................................................................
CEMacaque    ..H.......T...............................................................................
OBaboon      ..........T...............................................................................
Marmoset     .............................R.............A...........................................P..
BSqMonkey    .............................R.........................................................P..

Human        SKL
Chimpanzee   ...
Gorilla      ...
Orangutan    ...
Gibbon       ...
IRMacaque    .N.
CRMacaque    .N.
CEMacaque    .N.
OBaboon      ...
Marmoset     ...
BSqMonkey    ...
```

Green font highlighted in yellow: Human and Neandertal amino acid residue differ from chimpanzee.

SCP2

```
                       10        20        30        40        50        60        70        80        90
                        |         |         |         |         |         |         |         |         |
Human        MSSSPWEPATLRRVFVVGVGMTKFVKPGAENSRDYPDLAEEAGKKALADAQIPYSAVDQACVGYVFGDSTCGQRAIYHSLGMTGIPIINV
Chimpanzee   ..........................................................................................
Gorilla      ..........................................................................................
Orangutan    ....L....P.............................G..................................................
Gibbon       ....Q....P.............................G..................................................
IRMacaque    .........P...............R.............G...Q..............................................
CRMacaque    .........P...............R.............G...Q..............................................
CEMacaque    .........P...............R.............G...Q..............................................
OBaboon      .........P...............R.............G...Q...D..........................................
Marmoset     .F..LQ.L.P..............K..............R...Q..............................................
BSqMonkey    .F..LQ.R.P..............E..............R...Q.............E................................

                      100       110       120       130       140       150       160       170       180
                        |         |         |         |         |         |         |         |         |
Human        NNNCATGSTALFMARQLIQGGVAECVLALGFEKMSKGSLGIKFSDRTIPTDKHVDLLINKYGLSAHPVAPQMFGYAGKEHMEKYGTKIEH
Chimpanzee   ..........................................................................................
Gorilla      ..........................................................................................
Orangutan    ..................................N................................I......................
Gibbon       ...........................................L.........L....................................
IRMacaque    ....S................................................L.V..................................
CRMacaque    ....S................................................L.V..................................
CEMacaque    ....S................................................L.V..................................
OBaboon      ....S................................................L.V..................................
Marmoset     ....S.......V.....................E..G..T..L...S.L...L....................L.....I.........
BSqMonkey    ....S.......I.....................E..G.........N.L...L....D.....S.........L.....I.........

                      190       200       210       220       230       240       250       260       270
                        |         |         |         |         |         |         |         |         |
Human        FAKIGWKNHKHSVNNPYSQFQDEYSLDEVMASKEVFDFLTILQCCPTSDGAAAAILASEAFVQKYGLQSKAVEILAQEMMTDLPSSFEEK
Chimpanzee   .............................................................L............................
Gorilla      ..............................................................H...........................
Orangutan    ..............................................................H...........................
Gibbon       ..........................................................................................
IRMacaque    .................................K........................................................
CRMacaque    .................................K........................................................
CEMacaque    .................................K........................................................
OBaboon      .................................K........................................................
Marmoset     .................................K..E...............................P.....................
BSqMonkey    .................................K..................................P.....................

                      280       290       300       310       320       330       340       350       360
                        |         |         |         |         |         |         |         |         |
Human        SIIKMVGFDMSKEAARKCYEKSGLTPNDIDVIELHDCFSTNELLTYEALGLCPEGQGATLVDRGDNTYGGKWVINPSGGLISKGHPLGAT
Chimpanzee   ..........................................................................................
Gorilla      ..........................................................................................
Orangutan    ....V.....................................................................................
Gibbon       .V........................................................................................
IRMacaque    .V.............K..........................................................................
CRMacaque    .V........................................................................................
CEMacaque    .V........................................................................................
OBaboon      .V........................................................................................
Marmoset     .M.................K......S............A.................G................................
BSqMonkey    .M........................S............A.................G................................

                      370       380       390       400       410       420       430       440       450
                        |         |         |         |         |         |         |         |         |
Human        GLAQCAELCWQLRGEAGKRQVPGAKVALQHNLGIGGAVVVTLYKMGFPEAASSFRTHQIEAVPTSSASDGFKANLVFKEIEKKLEEEGEQ
Chimpanzee   ..........................................................................................
Gorilla      .........................................I................................................
Orangutan    .................................L...................S............S.......................
Gibbon       ..................................................T.......................................
IRMacaque    .................................L...........................A..N.........................
CRMacaque    .................................L...........................A..N.........................
CEMacaque    .................................L...........................A..N.........................
OBaboon      .................................L...........................A..N.........................
Marmoset     .................................L...........................A............................
BSqMonkey    .................E...............L........................................................
```

```
                    460       470       480       490       500       510       520       530       540
                      |         |         |         |         |         |         |         |         |
Human        FVKKIGGIFAFKVKDGPGGKEATWVVDVKNGKGSVLPNSDKKADCTITMADSDFLALMTGKMNPQSAFFQGKLKITGNMGLAMKLQNLQL
Chimpanzee   ......................................................................................S...
Gorilla      ..........................................................................................
Orangutan    ..........................................................................................
Gibbon       ..........................................................................................
IRMacaque    ..........................................................................................
CRMacaque    ..........................................................................................
CEMacaque    ..........................................................................................
OBaboon      ..........................................................................................
Marmoset     .....................................................L....................................
BSqMonkey    .....................................................L....................................

Human        QPGNAKL
Chimpanzee   .......
Gorilla      .......
Orangutan    .......
Gibbon       .......
IRMacaque    .......
CRMacaque    .......
CEMacaque    .......
OBaboon      .......
Marmoset     ...K...
BSqMonkey    ...K...
```

```
ACAA1                   10        20        30        40        50        60        70        80        90
                         |         |         |         |         |         |         |         |         |
Human           MQRLQVVLGHLRGPADSGWMPQAAPCLSGAPQASAADVVVVHGRRTAICRAGRGGFKDTTPDELLSAVMTAVLKDVNLRPEQLGDICVGN
Chimpanzee      ..........................................................................................
Gorilla         ....H.....................................................................................
Orangutan       ..........................................................................................
Gibbon          ..............................S...........................................................
IRMacaque       ...........T.RP...................................................................E........
CRMacaque       ...........T.RP...................................................................E........
CEMacaque       ...........T.RP...................................................................E........
OBaboon         ...........T.RP...................................................................E........
Marmoset        ...........T.QPHP..TL.....S..D...........................................Q................
BSqMonkey       .R.........A.QP.P..TL.....S..............................................Q................

                       100       110       120       130       140       150       160       170       180
                         |         |         |         |         |         |         |         |         |
Human           VLQPGAGAIMARIAQFLSDIPETVPLSTVNRQCSSGLQAVASIAGGIRNGSYDIGMACGVESMSLADRGNPGNITSRLMEKEKARDCLIP
Chimpanzee      ..........................................................................................
Gorilla         ..........................................................................................
Orangutan       ..........................................................................................
Gibbon          ..........................................................................................
IRMacaque       ..........................................................................................
CRMacaque       ..........................................................................................
CEMacaque       ..........................................................................................
OBaboon         ..........................................................................................
Marmoset        ..........................................................................................
BSqMonkey       ...........................S..............................................................

                       190       200       210       220       230       240       250       260       270
                         |         |         |         |         |         |         |         |         |
Human           MGITSENVAERFGISREKQDTFALASQQKAARAQSKGCFQAEIVPVTTTVHDDKGTKRSITVTQDEGIRPSTTMEGLAKLKPAFKKDGST
Chimpanzee      ..........................................................................................
Gorilla         ..........................................................................................
Orangutan       .........................................................................I................
Gibbon          .................................................IN.......................................
IRMacaque       ..........................................................................................
CRMacaque       ..........................................................................................
CEMacaque       ..........................................................................................
OBaboon         ..........................................................................................
Marmoset        ..........................................................................................
BSqMonkey       ................................................M.........................................

                       280       290       300       310       320       330       340       350       360
                         |         |         |         |         |         |         |         |         |
Human           TAGNSSQVSDGAAAILLARRSKAEELGLPILGVLRSYAVVGVPPDIMGIGPAYAIPVALQKAGLTVSDVDIFEINEAFASQAAYCVEKLR
Chimpanzee      ..........................................................................................
Gorilla         ..........................................................................................
Orangutan       ..........................................................................................
Gibbon          ..........................................................................................
IRMacaque       ........................................................I.................................
CRMacaque       ........................................................I.................................
CEMacaque       ........................................................I.................................
OBaboon         ........................................................I.................................
Marmoset        ..........................................................................................
BSqMonkey       ..........................................................................................

                       370       380       390       400       410       420
                         |         |         |         |         |         |
Human           LPPEKVNPLGGAVALGHPLGCTGARQVITLLNELKRRGKRAYGVVSMCIGTGMGAAAVFEYPGN
Chimpanzee      ................................................................
Gorilla         ................................................................
Orangutan       ................................................................
Gibbon          ................................................................
IRMacaque       ..S.............................................................
CRMacaque       ..S.............................................................
CEMacaque       ..S.............................................................
OBaboon         ..S.............................................................
Marmoset        ................................................V...............
BSqMonkey       ................................................................
```

PEX7

```
                      10        20        30        40        50        60        70        80        90
                       |         |         |         |         |         |         |         |         |
Human        MSAVCGGAARMLRTPGRHGYAAEFSPYLPGRLACATAQHYGIAGCGTLLILDPDEAGLRLFRSFDWNDGLFDVTWSENNEHVLITCSGDG
Chimpanzee   ..........T...............................................................................
Bonobo       ..........T...............................................................................
Gorilla      ..........T...............................................................................
Orangutan    ..........T...............................................................................
Gibbon       ..E.......T...............................................................................
IRMacaque    .......P..T..V.........................................S..................................
CRMacaque    .......P..T..V.........................................S..................................
CEMacaque    .......P..T..V.........................................S..................................
OBaboon      .......P..T..V.........................................S..................................
Marmoset     .N........T..V.....................A.................N.S..........S................V......
BSqMonkey    .N........T..V.....................A.................N.S..........S................V......

                     100       110       120       130       140       150       160       170       180
                       |         |         |         |         |         |         |         |         |
Human        SLQLWDTAKAAGPLQVYKEHAQEVYSVDWSQTRGEQLVVSGSWDQTVKLWDPTVGKSLCTFRGHESIIYSTIWSPHIPGCFASASGDQTL
Chimpanzee   ...........E..............................................................................
Bonobo       ...........E..............................................................................
Gorilla      ....................T.....................................................................
Orangutan    ....................T.....................................................................
Gibbon       .........T..........T.....................................................................
IRMacaque    ....................T.....................................................................
CRMacaque    ....................T.....................................................................
CEMacaque    ....................T.....................................................................
OBaboon      ....................T.....................................................................
Marmoset     ........R...........T.......................R....................NV.......................
BSqMonkey    ........R.......C...T...........................Y......NV.......................

                     190       200       210       220       230       240       250       260       270
                       |         |         |         |         |         |         |         |         |
Human        RIWDVKAAGVRIVIPAHQAEILSCDWCKYNENLLVTGAVDCSLRGWDLRNVRQPVFELLGHTYAIRRVKFSPFHASVLASCSYDFTVRFW
Chimpanzee   ...............G..........................................................................
Bonobo       .............................S............................................................
Gorilla      ..........................................................................................
Orangutan    ..........................................................................................
Gibbon       ..........................................................................................
IRMacaque    .............................S............................................................
CRMacaque    .............................S............................................................
CEMacaque    .............................S............................................................
OBaboon      .............................S............................................................
Marmoset     ....................................................................V.....................
BSqMonkey    ..........................................................................................

                     280       290       300       310       320
                       |         |         |         |         |
Human        NFSKPDSLLETVEHHTEFTCGLDFSLQSPTQVADCSWDETIKIYDPACLTIPA
Chimpanzee   .....................................................
Bonobo       .....................................................
Gorilla      .....................................................
Orangutan    .....................................................
Gibbon       .....................................................
IRMacaque    ......F..............................................
CRMacaque    ......F..............................................
CEMacaque    ......F..............................................
OBaboon      ......F..............................................
Marmoset     ......P..............................................
BSqMonkey    ......P..............................................
```

Green font highlighted in yellow: Human and Neandertal amino acid residue differ from chimpanzee.

PECR

```
                       10        20        30        40        50        60        70        80        90
                        |         |         |         |         |         |         |         |         |
Human        MASWAKGRSYLAPGLLQGQVAIVTGGATGIGKAIVKELLELGSNVVIASRKLERLKSAADELQANLPPTKQARVIPIQCNIRNEEEVNNL
Chimpanzee   ..........................................................................................
Gorilla      ..........................................................................................
Orangutan    ...........................................................G..............................
IRMacaque    .........C.................................................V..K.S....N....................
CRMacaque    .........C.................................................V..K.S....N....................
CEMacaque    .........C.................P...............................V..K.S....N....................
OBaboon      .........C....................................................K.S....N....................
Marmoset     .EG.V..K.F......E..................................F..........N.S....S..Q.........K.......

                      100       110       120       130       140       150       160       170       180
                        |         |         |         |         |         |         |         |         |
Human        VKSTLDTFGKINFLVNNGGGQFLSPAEHISSKGWHAVLETNLTGTFYMCKAVYSSWMKEHGGSIVNIIVPTKAGFPLAVHSGAARAGVYN
Chimpanzee   ..........................................................................................
Gorilla      ..........................................................................................
Orangutan    ......I.................L............................................SI.T.L...............
IRMacaque    ...........................R.........................N...............LA.......A...........
CRMacaque    ...........................R.........................N...............LA.......A...........
CEMacaque    ...........................R.........................N...............LA.......A...........
OBaboon      ....I......................R.........................N...............LA.......A...........
Marmoset     ......IY..............F..............V...............N....Q.........LV..T.V...A......E...S

                      190       200       210       220       230       240       250       260       270
                        |         |         |         |         |         |         |         |         |
Human        LTKSLALEWACSGIRINCVAPGVIYSQTAVENYGSWGQSFFEGSFQKIPAKRIGVPEEVSSVVCFLLSPAASFITGQSVDVDGGRSLYTH
Chimpanzee   .....................................................................................G....
Gorilla      .....................................................................................G....
Orangutan    .............V.....................Y......E...............................................
IRMacaque    .............V........I......A.....F...L..S.........................................QC...Y
CRMacaque    .............V........I......A.....F...L..S.........................................QC...Y
CEMacaque    .............V........I......A.....F...L..S.........................................QC...Y
OBaboon      .............V........I......A.....F...L..S....T....................................QC....
Marmoset     ......F......V........F...P..A.....S...L..SF..............I.........................Q..FS.

                      280       290       300
                        |         |         |
Human        SYEVPDHDNWPKGAGDLSVVKKMKETFKEKAKL
Chimpanzee   .................................
Gorilla      ....................E............
Orangutan    ...I.................R...........
IRMacaque    ...........E.........R..VS..D....
CRMacaque    ...........E.........R..VS..D....
CEMacaque    ...........E.........R..AS..D....
OBaboon      ...........E.........R..AS..D....
Marmoset     WC.L.......E.............S..Q....
```
