# Supplementary material for: Diverse captive non-human primates with phytanic acid-deficient diets rich in plant products have substantial phytanic acid levels in their red blood cells
Source: Lipids Health Dis. 2013 Feb 4;12:10. doi: 10.1186/1476-511X-12-10 (PMC3571895; doi:10.1186/1476-511X-12-10)
Supplement: Additional file 6 — Results of PAML likelihood ratio tests for selection. Results of PAML likelihood ratio tests for selection are provided. [file 1476-511X-12-10-S6.docx]

**Additional File 6.** Results of PAML likelihood ratio tests for selection.

| **Test/Model** | *ACAA1* | *ACOX2* | *ACOX3* | *ACSL1* | *ALDH3A2* | *AMACR* | *EHHADH* | *HACL1* | *PEX7* | *PHYH* | *SCP2* | *SLC27A2* | | *PECR* |
| --- | --- | --- | --- | --- | --- | --- | --- | --- | --- | --- | --- | --- | --- | --- |
| **Branch Model Test** |  | | | | | | | | | | | | | |
| M7 | -2272.07 | -4100.99 | -4773.62953 | -4164.98 | -2973.73 | -2639.44 | -4282.48 | -3298.87 | -1820.4 | -2451.84 | -3118.21 | | -3758.8 | -1855.0 |
| M8 | -2272.07 | -4096.17 | -4773.62953 | -4163.37 | -2972.89 | -2638.42 | -4282.47 | -3298.05 | -1820.12 | -2451.54 | -3115.18 | | -3757.36 | -1847.94 |
| DF | 2 | 2 | 2 | 2 | 2 | 2 | 2 | 2 | 2 | 2 | 2 | | 2 | 2 |
| 2×LLR | -4E-05 | 9.63786* | 0 | 3.2121 | 1.66414 | 2.02496 | 0.02076 | 1.65028 | 0.5641 | 0.60168 | 6.0486* | | 2.88588 | 16.1113** |
|  | | | | | | | | | | | | | | |
| **Site Model Test** |  | | | | | | | | | | | | | |
| Model 0 (1 dN/dS) | -2339.31 | -4214.66 | -4852.41244 | -4229.79 | -3021.07 | -2702.57 | -4347.53 | -3379.39 | -1850.61 | -2485.25 | -3209.66 | | -3840.04 | -1866.05 |
| Model 1 (vary dN/dS) | -2336.02 | -4206.97 | -4838.64343 | -4221.96 | -3013.3 | -2682.66 | -4339.83 | -3371.24 | -1839.66 | -2474.06 | -3200.5 | | -3828.22 | -1861.41 |
| DF | 14 | 18 | 18 | 14 | 14 | 22 | 16 | 16 | 18 | 22 | 16 | | 16 | 14 |
| 2×LLR | 6.57296 | 15.38808 | 27.53802 | 15.65004 | 15.54232 | 39.82416* | 15.41674 | 16.3047 | 21.9009 | 22.38468 | 18.32032 | | 23.64266 | 9.2645 |
|  | | | | | | | | | | | | | | |
| **Branch-Site Model Test** |  | | | | | | | | | | | | | |
| Chimpanzee Branch |  |  |  |  |  |  |  |  |  |  |  |  |  |  |
| Model C | -2344.76 | -4204.87 | -4840.53131 | -4260.87 | -3016.75 | -2699.63 | -4345.8 | -3418.25 | -1853 | -2471.7 | -3195.45 | | -3836.78 | -1855.98 |
| Null Model | -2344.63 | -4202.37 | -4840.43034 | -4259.9 | -3016.68 | -2698.72 | -4345.31 | -3418.25 | -1852.73 | -2469.19 | -3192.79 | | -3833.35 | -1855.98 |
| DF | 3 | 3 | 3 | 3 | 3 | 3 | 3 | 3 | 3 | 3 | 3 | | 3 | 3 |
| 2×LLR | 0.26842 | 4.99528 | 0.20194 | 1.93126 | 0.13526 | 1.8222 | 0.97506 | 0.0 | 0.53958 | 5.0138 | 5.33054 | | 6.8599 | 0.0 |
|  | | | | | | | | | | | | | | |
| **Branch-Site Model Test** |  | | | | | | | | | | | | | |
| Human Branch |  |  |  |  |  |  |  |  |  |  |  |  |  |  |
| Model C | -2344.76 | -4204.87 | -4840.53131 | -4260.87 | -3016.75 | -2699.63 | -4345.8 | -3418.25 | -1853 | -2471.7 | -3195.45 | | -3836.78 | -1855.98 |
| Null Model | -2344.21 | -4204.72 | -4840.04161 | -4260.73 | -3016.57 | -2699.09 | -4344.83 | -3418.25 | -1850.97 | -2471.32 | -3195.45 | | -3836.72 | -1855.98 |
| DF | 3 | 3 | 3 | 3 | 3 | 3 | 3 | 3 | 3 | 3 | 3 | | 3 | 3 |
| 2×LLR | 1.1102 | 0.29768 | 0.9794 | 0.28062 | 0.3439 | 1.07816 | 1.94252 | 0.0 | 4.06302 | 0.74454 | 0 | | 0.12186 | 0.0 |

We estimated gene phylogenies using a Bayesian approach [11] under a codon substitution model in which mutation rates follow a gamma+ invariant distribution and allows for codons to have 1/3 different ratios of non-synonymous to synonymous (dN/dS) mutation rates. These trees were used in tests of variation in the dN/dS value using the PAML program (Yang Z: **PAML 4: phylogenetic analysis by maximum likelihood**. *Mol Biol Evol* 2007, **24**:1586-1591). To test whether some amino acid sites had been evolving with dN/dS >1, we used a null model in which dN/dS values vary among sites under a Beta distribution (all values vary between zero and one), and an alternative model in which the distribution also includes a fraction of sites with a dN/dS >1 (contrast of PAML models 7 and 8). If the null model is true, the likelihood-ratio statistic should follow a χ2 with 2 degrees of freedom (DF). To test for significant variation in dN/dS values among the branches, we estimated dN/dS values for individual branches using PAML (NSsites=0, model=1) and contrasted this with the base model in which all branches had equivalent dN/dS value (NSsites=0, model=0). To investigate whether dN/dS values varied significantly over the phylogeny, we allowed all branches on the foreground ape clade to have a different dN/dS value than the remainder of the tree. Using Clade model C, we tested whether adding this partition led to a significantly better fit to the data than a model in which all branches shared the same dN/dS ratio. This test was also conducted with the human branch isolated as the foreground clade. PAML 4.5 likelihood ratio tests were used in this analysis. For each gene the likelihoods are shown for two nested models, together with the degrees of freedom and the level of significance. For each model the equilibrium codon frequencies were specified to be equal to the actual observed frequencies (PAML setting CodonFreq =2). The Branch Model Test contrasts a model in which all branches of the phylogeny have the same dN/dS ratio with one in which each branch has its own dN/dS ratio. The number of degrees of freedom is equal to the number of branches on the tree, minus 1. The Site Model Test contrasts a model (model M7) in which the dN/dS ratio for an amino acid is sampled from a beta distribution (i.e. all dN/dS values <1) with a model (M8) in which some fraction of sites experience dN/dS >1. There are 2 DF with this test. The Branch Site Model Test contrasts a model in which dN/dS values can vary among sites and user-specified “foreground” branches in the phylogeny (model C) with a model in which all branches have the same distribution of dN/dS values. There are 3 DF and results are shown for tests in which the foreground branches include all branches among ape sequences (including lesser and great apes) and for tests in which only the human branch is the foreground branch. **P* <0.05, ***P* <0.01.
